# Biomaterial-mediated remodelling of the inflammatory microenvironment: a pH/ROS-responsive EGCG–metformin hydrogel for infected diabetic wound regeneration

**DOI:** 10.3389/fcell.2026.1913797

**Published:** 2026-08-07

**Authors:** Rui Zhang, Suk Fei Tan, Ye Wang, Wenqin Zhang, Junxue Wu, Chao Zhang

**Affiliations:** 1 Department of Orthopedics, Affiliated Hospital of North Sichuan Medical College, Nanchong, Sichuan, China; 2 School of Graduate Studies, Post Graduate Centre, Management and Science University, Shah Alam, Selangor, Malaysia; 3 School of Pharmacy, Management and Science University, Shah Alam, Selangor, Malaysia; 4 Trauma Medical Center, Department of Orthopaedic Surgery, West China Hospital, Sichuan University, Chengdu, China; 5 Department of Orthopaedics, West China Hospital, Sichuan University, Chengdu, China; 6 Surgical Center, Affiliated Hospital of North Sichuan Medical College, Nanchong, Sichuan, China

**Keywords:** angiogenesis, diabetic chronic wound, epigallocatechin gallate, macrophage polarisation, metformin, oxidative stress, pH/ROS-responsive drug release, smart hydrogel

## Abstract

**Introduction:**

Diabetic chronic wounds resist healing because persistent bacterial infection, excessive reactive oxygen species (ROS), unresolved pro-inflammatory responses, impaired angiogenesis, and defective tissue remodelling act simultaneously and reinforce one another. We therefore developed an injectable, microenvironment-responsive nanocomposite hydrogel, AP@EM-gel, to target these interconnected pathological processes.

**Methods:**

AP@EM-gel was constructed from dopamine-grafted alginate (Alg-DA), phenylboronic-acid-modified ε-poly-L-lysine (EPBA), and co-assembled epigallocatechin gallate–metformin nanoparticles (EGCG-MET NPs). Its physicochemical properties, pH/ROS-responsive drug release, antibacterial and antioxidant activities, cytocompatibility, pro-angiogenic effects, and macrophage-modulating capacity were evaluated *in vitro*. Therapeutic efficacy was further assessed in a streptozotocin-induced diabetic rat model of Staphylococcus aureus-infected full-thickness wounds.

**Results:**

Dynamic boronate-ester crosslinking produced a self-healing and injectable network that released approximately 73% of EGCG and 68% of metformin under combined pH 6.4 and H_2_O_2_ conditions, compared with approximately 38% and 36%, respectively, at pH 7.4. AP@EM-gel achieved antibacterial rates of approximately 93% against *S. aureus* and 91% against *Escherichia coli*, exhibited broad-spectrum radical-scavenging activity, and showed favourable cyto- and haemocompatibility. It restored VEGF and bFGF expression in oxidatively stressed endothelial cells and promoted macrophage repolarisation toward the reparative M2 phenotype. *In vivo*, AP@EM-gel produced near-complete wound closure by day 14 and improved bacterial clearance, re-epithelialisation, collagen organisation, angiogenesis, and inflammatory resolution compared with the commercial dressing.

**Discussion:**

AP@EM-gel simultaneously interrupts infection, oxidative stress, dysregulated macrophage polarisation, and impaired angiogenesis. This pathology-responsive, multi-target hydrogel represents a promising smart dressing for infected diabetic wound regeneration.

## 1 Introduction

Diabetic chronic wounds are among the most intractable problems in contemporary wound care, imposing a heavy and growing burden on patients and health systems worldwide. Unlike acute wounds, which progress in an orderly fashion through haemostasis, inflammation, proliferation and remodelling, diabetic wounds become trapped in a prolonged inflammatory state and frequently fail to close, predisposing patients to infection, amputation and death (Sen, 2019; Armstrong et al., 2023). This chronicity does not arise from a single defect but from the concurrent operation of several mutually reinforcing pathological processes: persistent bacterial colonisation and biofilm formation, pathological accumulation of reactive oxygen species (ROS), sustained dominance of pro-inflammatory M1 macrophages, impaired angiogenesis, and arrested extracellular-matrix remodelling (Deng et al., 2023; Aitcheson et al., 2021; Monaghan et al., 2023). Each of these drivers amplifies the others, so that infection fuels oxidative stress, oxidative stress sustains M1 inflammation, inflammation suppresses angiogenesis, and the resulting hypoxia further promotes infection and oxidative damage (Kunkemoeller and Kyriakides, 2017; Jain et al., 2025). Effective treatment therefore requires simultaneous intervention at multiple nodes of this loop rather than correction of any one abnormality in isolation (Cai et al., 2023; Zhao et al., 2024).

Hydrogel dressings have attracted intense interest because their hydrated, tissue-like networks maintain a moist healing environment and can be engineered to carry bioactive payloads. Yet most clinically available dressings—including widely used commercial products such as the polyurethane-based Hydrosorb® hydrogel—function largely as passive moisture managers: they absorb exudate and protect the wound but possess no intrinsic antibacterial, antioxidant, immunomodulatory or pro-angiogenic activity (Dong and Guo, 2021; Wang et al., 2024). Even advanced research dressings that incorporate a single antibacterial agent, antioxidant or growth factor address only one dimension of the multifactorial pathology and leave the remaining drivers of chronicity intact. A further limitation of conventional drug-loaded systems is that they release their cargo passively and continuously, depleting the payload regardless of where, or how severe, the pathology actually is. There is consequently a clear and unmet need for a single platform that is at once injectable and wound-adaptive, intrinsically antibacterial, broadly antioxidant, immunomodulatory and pro-angiogenic, and capable of matching drug delivery to the biochemical state of the wound (Yu et al., 2024; Wang et al., 2024).

**FIGURE 1:**
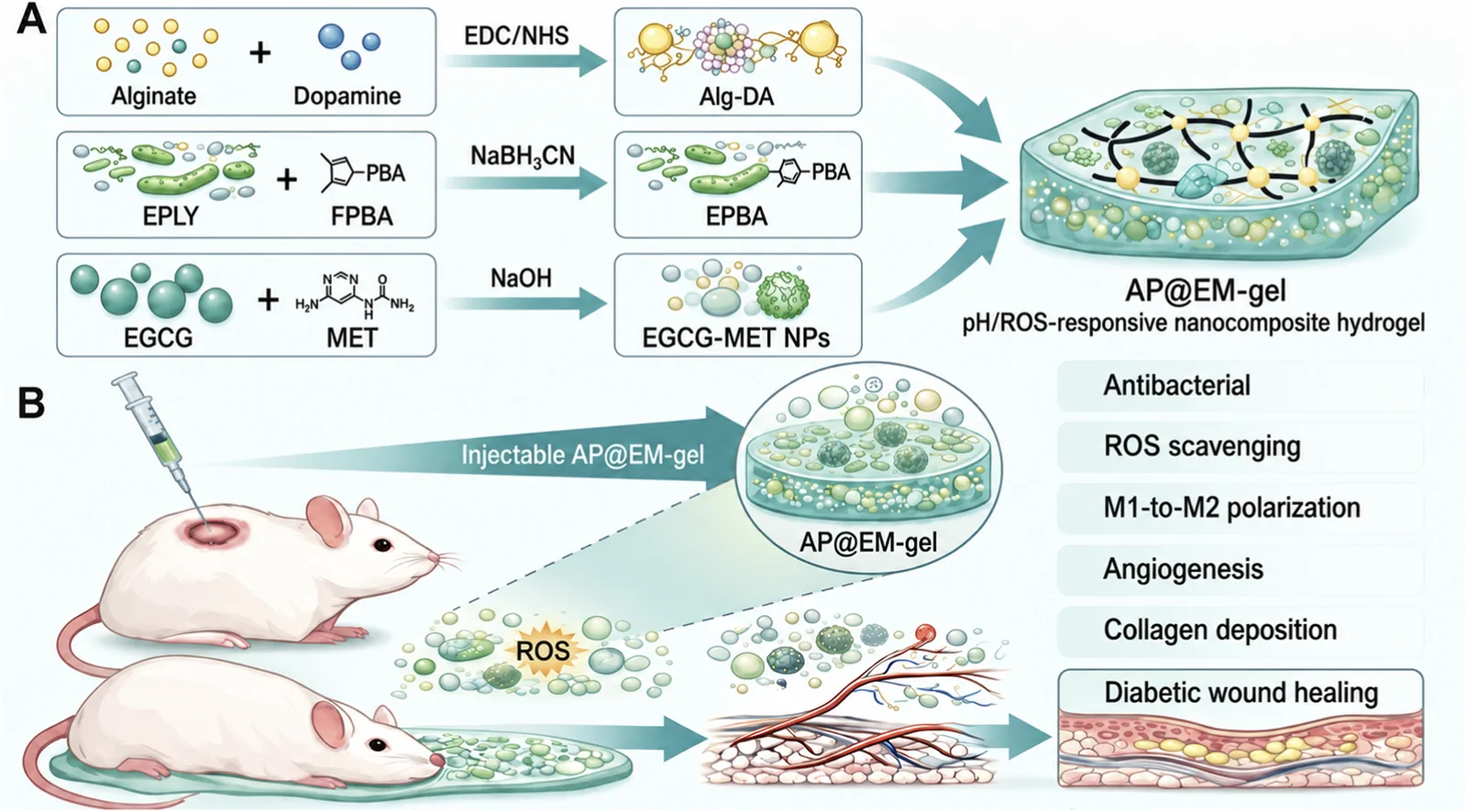
Schematic of the design and multi-target mechanism of AP@EM-gel. **(A)** Synthesis of the three building blocks—dopamine-grafted alginate (Alg-DA) by EDC/NHS coupling, phenylboronic-acid-modified ε-poly-L-lysine (EPBA) by NaBH_3_CN reductive amination from EPLY and FPBA, and co-assembled EGCG–metformin nanoparticles (EGCG-MET NPs) under alkaline conditions—which together form the AP@EM-gel network. **(B)** Injectable application of AP@EM-gel in a diabetic infected wound and the resulting multifunctional effects (antibacterial activity, ROS scavenging, M1-to-M2 macrophage polarisation, angiogenesis and collagen deposition), driving inflammation alleviation and tissue regeneration. Created with BioRender.com. No generative AI was used to generate Figure 1.

To meet this need we rationally designed AP@EM-gel, an injectable, microenvironment-responsive nanocomposite hydrogel that engages all four pathological barriers through three functionally complementary components (Figure 1). The platform is named for its constituents: “AP” denotes the Alg-DA/EPBA boronate-ester network, “EM” the EGCG–MET nanoparticle payload, and “@” the loading of that payload within the network. Dopamine-grafted alginate (Alg-DA) provides the catechol-bearing structural backbone, conferring mussel-inspired wet-tissue adhesion and supplying the catechol partners for dynamic crosslinking (Scognamiglio et al., 2016; Quan et al., 2019). Phenylboronic-acid-modified ε-poly-L-lysine (EPBA) serves a dual role: its boronic-acid groups form reversible boronate-ester bonds with Alg-DA catechols to build a self-healing, injectable and stimulus-responsive network, while its cationic polylysine backbone disrupts bacterial membranes through a non-antibiotic, resistance-independent electrostatic mechanism (Hyldgaard et al., 2014; Yang et al., 2021). Co-assembled epigallocatechin gallate–metformin nanoparticles (EGCG-MET NPs) constitute the therapeutic payload, combining the potent radical-scavenging, anti-inflammatory and pro-angiogenic activity of the green-tea polyphenol EGCG with the AMPK-mediated metabolic, anti-inflammatory and vascular-protective actions of metformin (Mokra et al., 2022; Martin-Montalvo et al., 2013).

Although EGCG-based and metformin-loaded dressings have each been reported individually, the present design is distinguished by co-assembling the two agents into a single nanoparticle and coupling them to a pathology-gated boronate-ester network, so that a complementary antioxidant–anti-inflammatory payload is delivered on demand rather than from two separately loaded, passively releasing carriers. Critically, the boronate-ester network hydrolyses preferentially under the acidic and oxidative conditions that characterise the infected diabetic wound bed, so that AP@EM-gel releases its payload on demand and in proportion to local pathological severity (Hong et al., 2018; Deng et al., 2024). We hypothesised that this multi-target, microenvironment-responsive design would outperform a passive commercial dressing across the full spectrum of wound-healing outcomes. We tested this hypothesis through systematic physicochemical characterisation, a panel of *in vitro* biological assays, and an *in vivo* study in a streptozotocin-induced diabetic rat model of *Staphylococcus* aureus-infected full-thickness wounds.

## 2 Materials and methods

### 2.1 Materials

Sodium alginate (Alg), dopamine hydrochloride (DA), ε-poly-L-lysine (EPLY), 4-formylphenylboronic acid (FPBA), 1-ethyl-3-(3-dimethylaminopropyl)carbodiimide hydrochloride (EDC), N-hydroxysuccinimide (NHS), 2-morpholinoethanesulfonic acid (MES) and sodium cyanoborohydride (NaBH_3_CN) were obtained from commercial suppliers (Shanghai, China). Epigallocatechin gallate (EGCG) and metformin hydrochloride (MET) were purchased from McLean Biochemical Technology. *S. aureus* (ATCC 25923) and *E. coli* (ATCC 25922) were used as model pathogens. Mouse fibroblasts (L929), murine macrophages (RAW264.7) and human umbilical vein endothelial cells (HUVECs) were used for cell studies. Hydrosorb®, a polyurethane-based hydrogel sheet widely used in clinical practice, was selected as the commercial comparator because it is representative of the passive, moisture-managing dressings that dominate current diabetic-wound care and thus provides a clinically meaningful benchmark rather than a sham control.

L929 fibroblasts and RAW264.7 macrophages were cultured in high-glucose DMEM supplemented with 10% fetal bovine serum and 1% penicillin–streptomycin, and HUVECs in complete endothelial cell medium (5% fetal bovine serum, 1% endothelial cell growth supplement and 1% penicillin–streptomycin); all cells were maintained at 37 °C in a humidified atmosphere of 5% CO_2_ and used in the logarithmic growth phase.

### 2.2 Synthesis of Alg-DA, EPBA and EGCG-MET nanoparticles

Alg-DA. Catechol groups were grafted onto alginate by EDC/NHS-mediated amide coupling. Alginate was dissolved in MES buffer (0.1 M, pH 5.5); carboxyl groups were activated with EDC and NHS for 1 h, after which dopamine hydrochloride was added and the reaction proceeded for 24 h at room temperature, protected from light. The product was dialysed (MWCO 2 kDa) against water for 5 days and freeze-dried. Grafting was confirmed by ^1^H NMR.

EPBA. Phenylboronic-acid groups were introduced onto EPLY by reductive amination. FPBA in methanol was added dropwise to an aqueous EPLY solution and allowed to form a Schiff base for 8 h; NaBH_3_CN was then added and the mixture stirred overnight to reduce the imine to a stable secondary amine. The product was dialysed (MWCO 1 kDa), centrifuged and freeze-dried, and verified by ^1^H NMR.

EGCG-MET NPs. EGCG and MET were co-dissolved in water at a fixed mass ratio of 75 mg EGCG to 55 mg MET (≈1.4:1) in 25 mL deionised water, and the pH was raised to alkaline with 250 μL of 1 M NaOH (24 h at room temperature); under these conditions EGCG self-polymerised through oxidative coupling of its catechol and galloyl groups while MET was incorporated through Schiff-base and Michael-addition reactions with EGCG quinone intermediates. Nanoparticles were collected by centrifugation and characterised by the Tyndall effect, UV-Vis spectrophotometry and dynamic light scattering/ zeta potential.

### 2.3 Fabrication of AP-gel and AP@EM-gel

The base AP hydrogel was formed by boronate-ester condensation between EPBA and Alg-DA. An EPBA solution (5% w/v) was added to an Alg-DA solution (3% w/v) at a 1:2 volume ratio (EPBA:Alg-DA); gelation was confirmed by the inversion test and the network by FTIR. To prepare the drug-loaded AP@EM-gel, EGCG-MET NPs (final concentration 1 mg/mL) were dispersed in the Alg-DA solution before crosslinking. The Alg-DA:EPBA ratio and nanoparticle loading were optimised for gelation, mechanical integrity, injectability and biocompatibility. Hydrogel microstructure was examined by scanning electron microscopy and rheological behaviour by frequency- and strain-sweep tests.

### 2.4 pH/ROS-responsive drug release

Freeze-dried AP@EM-gel was sealed in dialysis bags (MWCO 3.5 kDa) and immersed in four media simulating distinct wound conditions: PBS pH 7.4 (normal tissue), PBS pH 6.4 (acidic infected/ischaemic bed), PBS pH 7.4 + 1 mM H_2_O_2_ (oxidative stress), and PBS pH 6.4 + 1 mM H_2_O_2_ (combined pathological condition). Media were sampled at intervals up to 72 h under sink conditions at 37 °C, and EGCG and MET were quantified by UV-Vis at 272 nm and 232 nm, respectively, against calibration curves (*R* ^2^ ≥ 0.999).

All release experiments were performed in triplicate (n = 3); at each sampling time an equal volume of fresh, pre-warmed medium was replenished to maintain sink conditions, and cumulative release was corrected for the volume withdrawn at preceding time points.

### 2.5 *In vitro* antibacterial and antioxidant evaluation

Antibacterial activity against *S. aureus* and *E. coli* was assessed by plate colony counting after 3 h co-culture of bacterial suspensions with hydrogel extracts (Blank, AP-gel, AP@EM-gel), and ultrastructural damage was visualised by SEM. Antioxidant capacity was characterised by five complementary assays: DPPH and ABTS radical scavenging, H_2_O_2_ scavenging (Ti(SO_4_)_2_ method), hydroxyl-radical scavenging (salicylic-acid probe/ Fenton reaction) and superoxide-anion scavenging (WST-8/ SOD kit).

For the antibacterial assay, bacterial suspensions were adjusted to ≈ 1 × 10^6^ CFU/mL and co-cultured with hydrogel extracts for 3 h at 37 °C; serial ten-fold dilutions were then plated on LB agar and viable colonies were counted after overnight incubation. The antibacterial rate (%) was calculated relative to the Blank group. All antibacterial and antioxidant assays were performed in triplicate (n = 3).

### 2.6 Pro-angiogenic activity, biocompatibility and immunomodulation

Pro-angiogenic activity was evaluated in HUVECs subjected to oxidative stress (100 μmol/L H_2_O_2_) by immunofluorescence for VEGF and bFGF across Control, H_2_O_2_, AP-gel and AP@EM-gel groups. Cytocompatibility was assessed in L929 fibroblasts by CCK-8 and Calcein-AM/PI live/dead staining, haemocompatibility by an erythrocyte haemolysis assay (against the ISO 10993–4 5% threshold), and migration by a scratch-wound assay. Immunomodulation was studied in LPS-stimulated RAW264.7 macrophages by CD206 (M2)/ iNOS (M1) immunofluorescence, flow cytometry for CD206+ and CD86+ proportions, and RT-qPCR for IL-10 and TNF-α mRNA.

HUVECs were seeded at 5 × 10^4^ cells/well on 35 mm glass-bottom dishes; L929 cells at 5 × 10^3^ cells/well in 96-well plates; and RAW264.7 cells at 5 × 10^4^ (immunofluorescence), 2 × 10^5^ (flow cytometry) or 5 × 10^5^ (RT-qPCR) cells/well, with a 24 h pre-incubation before treatment. For the inflammatory model, macrophages were primed with LPS (100 ng/mL, 24 h) and then treated with hydrogel extract (1 mg/mL) for 48 h. Flow-cytometry staining used FITC-conjugated anti-CD86 (1:100) and PE-conjugated anti-CD206 (1:100) antibodies. Hydrogel extracts were prepared at 100 mg/mL (37 °C, 24 h) and filtered (0.22 μm); haemolysis was calculated against Triton X-100 (positive) and PBS (negative) controls. All *in vitro* biological assays were performed in triplicate (n = 3).

### 2.7 *In vivo* diabetic infected wound model

All animal procedures were approved by the Animal Ethics Committee of West China Hospital, Sichuan University (approval No. 20250120003) and followed institutional guidelines and the 3R principles. Diabetes was induced in male Sprague-Dawley rats by a single tail-vein injection of streptozotocin (STZ, 65 mg/kg); animals with fasting blood glucose ≥ 16.7 mmol/L on two consecutive measurements were included. Full-thickness dorsal wounds (10 mm diameter) were created and inoculated with *S. aureus* (1 × 10^8^ CFU). Twenty-four hours later, wounds were assigned to four groups (Blank, Hydrosorb®, AP-gel, AP@EM-gel; ≈ 100 μL applied) and dressings were changed every 2 days over 14 days. Wound contraction was quantified by digital planimetry at days 0, 3, 7, 10 and 14; day-3 bacterial burden was measured by tissue CFU counting; and tissue collected at days 7 and 14 was analysed by H&E and Masson’s trichrome staining, CD31/α-SMA and CD206/iNOS immunofluorescence, and IL-10/TNF-α immunohistochemistry.

Rats (n = 6 per group) were randomly allocated to treatment groups. Full-thickness wounds were created under isoflurane anaesthesia (3% induction, 1.5% maintenance) after dorsal hair removal and 70% ethanol disinfection, using a sterile biopsy punch; animals were euthanised at the defined endpoints by isoflurane overdose. Wound areas were measured from calibrated digital photographs using ImageJ, and all histological and immunostaining quantifications were performed by an observer blinded to group allocation. For tissue CFU counting, wound tissue (≈100 mg) was aseptically excised, homogenised in 1 mL sterile PBS, serially diluted (10^−1^–10^−4^) and plated on LB agar (37 °C, 24 h).

### 2.8 Statistical analysis

Data are expressed as mean ± standard deviation. Comparisons among groups used one-way ANOVA with Tukey’s *post hoc* test; time-course data used two-way ANOVA (GraphPad Prism). p < 0.05 was considered significant (*p < 0.05, **p < 0.01, ***p < 0.001, ****p < 0.0001).

## 3 Results

### 3.1 Synthesis, assembly and pH/ROS-responsive release of AP@EM-gel

**FIGURE 2:**
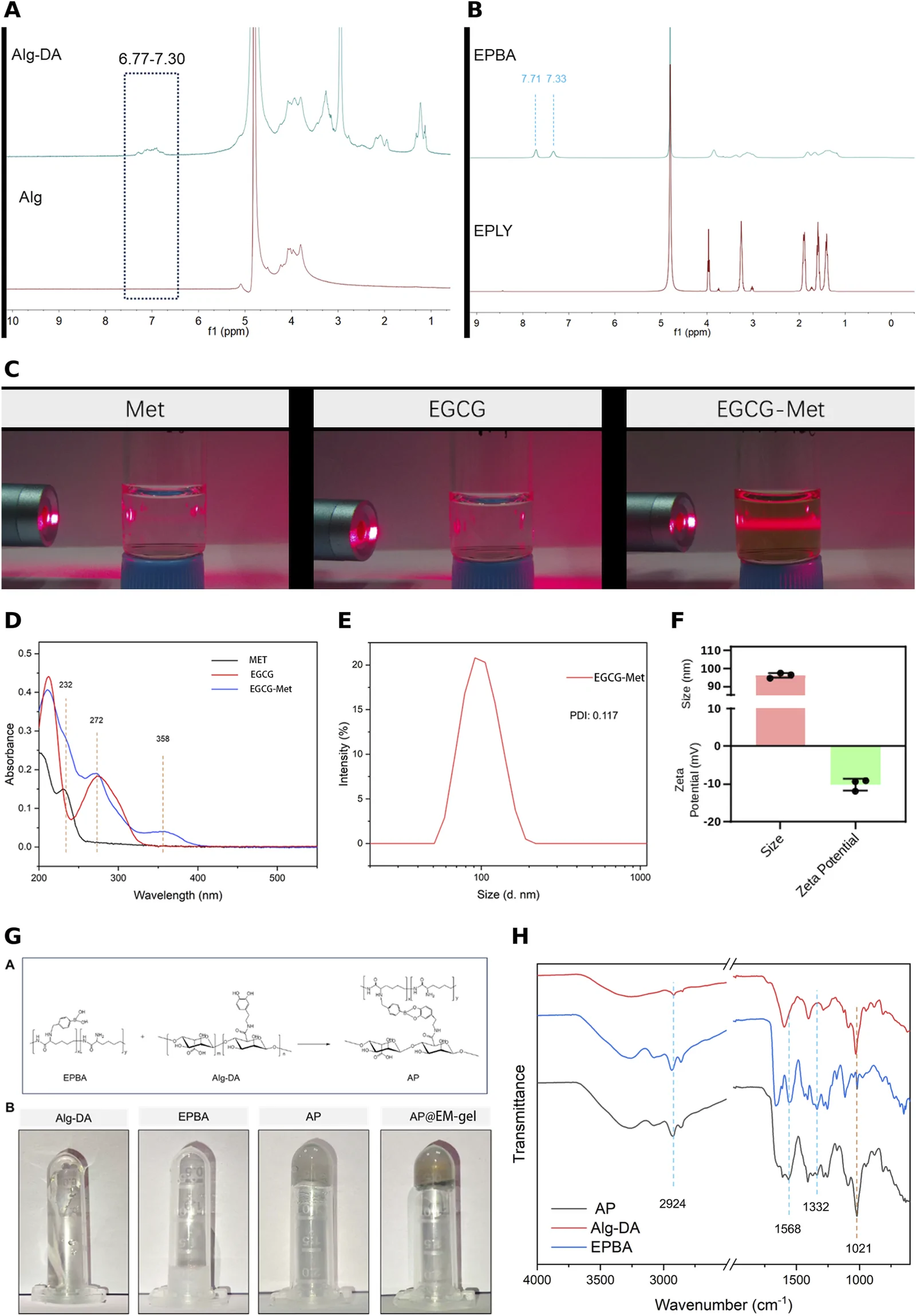
Physicochemical characterisation of AP@EM-gel. **(A)** ^1^H NMR spectra of alginate and Alg-DA. **(B)** ^1^H NMR spectra of EPLY and EPBA. **(C)** Tyndall effect of MET, EGCG and EGCG-MET NPs. **(D)** UV-Vis spectra of MET, EGCG and EGCG-MET NPs. **(E)** Hydrodynamic size distribution of EGCG-MET NPs. **(F)** Mean particle size and zeta potential of EGCG-MET NPs. **(G)** Synthesis scheme and inversion test of Alg-DA, EPBA, AP-gel and AP@EM-gel. **(H)** FTIR spectra of AP-gel, Alg-DA and EPBA. Data are mean ± SD (n = 3).

The three building blocks were synthesised and confirmed sequentially. ^1^H NMR of Alg-DA showed new aromatic signals at 6.77–7.30 ppm, absent in unmodified alginate, confirming amide coupling of dopamine to the alginate backbone (Figure 2A); EPBA showed two new aromatic signals at 7.33 and 7.71 ppm relative to EPLY, confirming phenylboronic-acid grafting (Figure 2B). EGCG-MET NPs displayed the EGCG aromatic peak at 272 nm, the MET peak at 232 nm, and a new red-shifted band at 358 nm arising from extended π–π conjugation during alkaline EGCG self-polymerisation (Figure 2D), together confirming co-assembly of both agents. The nanoparticles were uniform and colloidally stable, with a mean hydrodynamic diameter of 96.1 ± 1.1 nm and a narrow size distribution (Figure 2E); a clear Tyndall light path further verified their colloidal nature (Figure 2C), and the zeta potential of −10.13 ± 1.2 mV (Figure 2F) reflected their phenolic-hydroxyl-rich surface.

**FIGURE 3:**
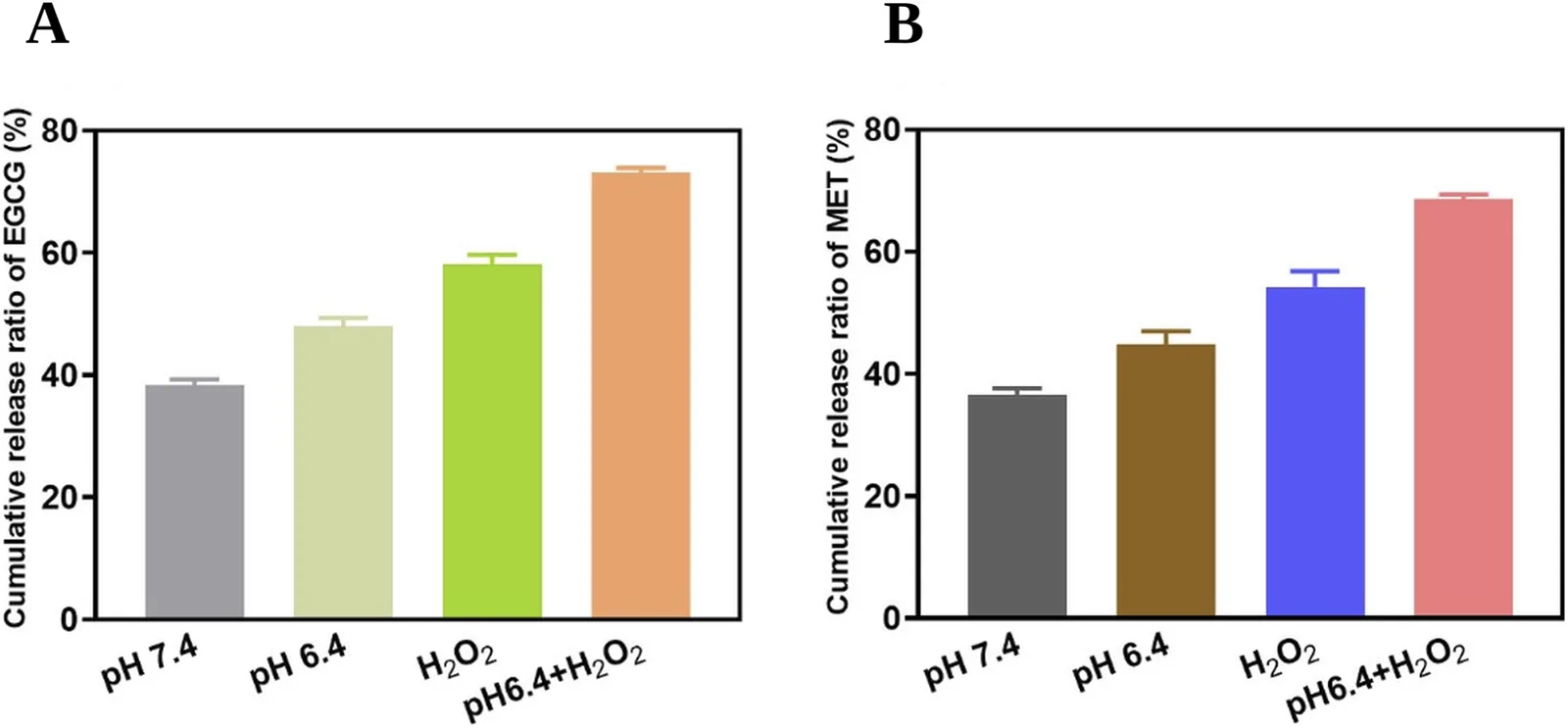
pH/ROS-responsive drug release. Cumulative release of **(A)** EGCG and **(B)** MET under four simulated microenvironmental conditions (pH 7.4, pH 6.4, pH 7.4 + 1 mM H_2_O_2_, and pH 6.4 + 1 mM H_2_O_2_). Data are mean ± SD (n = 3).

Mixing Alg-DA and EPBA produced a self-supporting gel that resisted flow on inversion, whereas the precursor solutions flowed freely (Figure 2G). FTIR of the AP network showed EPBA bands at 2924 cm^−1^ (CH2), 1568 cm^−1^ (aromatic C=C) and 1332 cm^−1^ (B–O), together with an enhanced Alg-DA C–O band at 1021 cm^−1^, confirming boronate-ester crosslinking (Figure 2H). Drug release was strongly microenvironment-dependent (Figure 3): cumulative EGCG release rose from ≈ 38% at pH 7.4 to ≈ 48% at pH 6.4, ≈ 58% under H_2_O_2_, and ≈ 73% under combined pH 6.4 + H_2_O_2_, with MET following a parallel profile (≈36%, 45%, 54%, and 68%, respectively; p < 0.05 among conditions). The hydrogel thus retained its payload under physiological conditions but released progressively more drug as acidity and oxidative stress increased, matching delivery to pathological severity.

### 3.2 *In vitro* antibacterial, antioxidant, angiogenic, cytocompatibility and immunomodulatory performance

**FIGURE 4:**
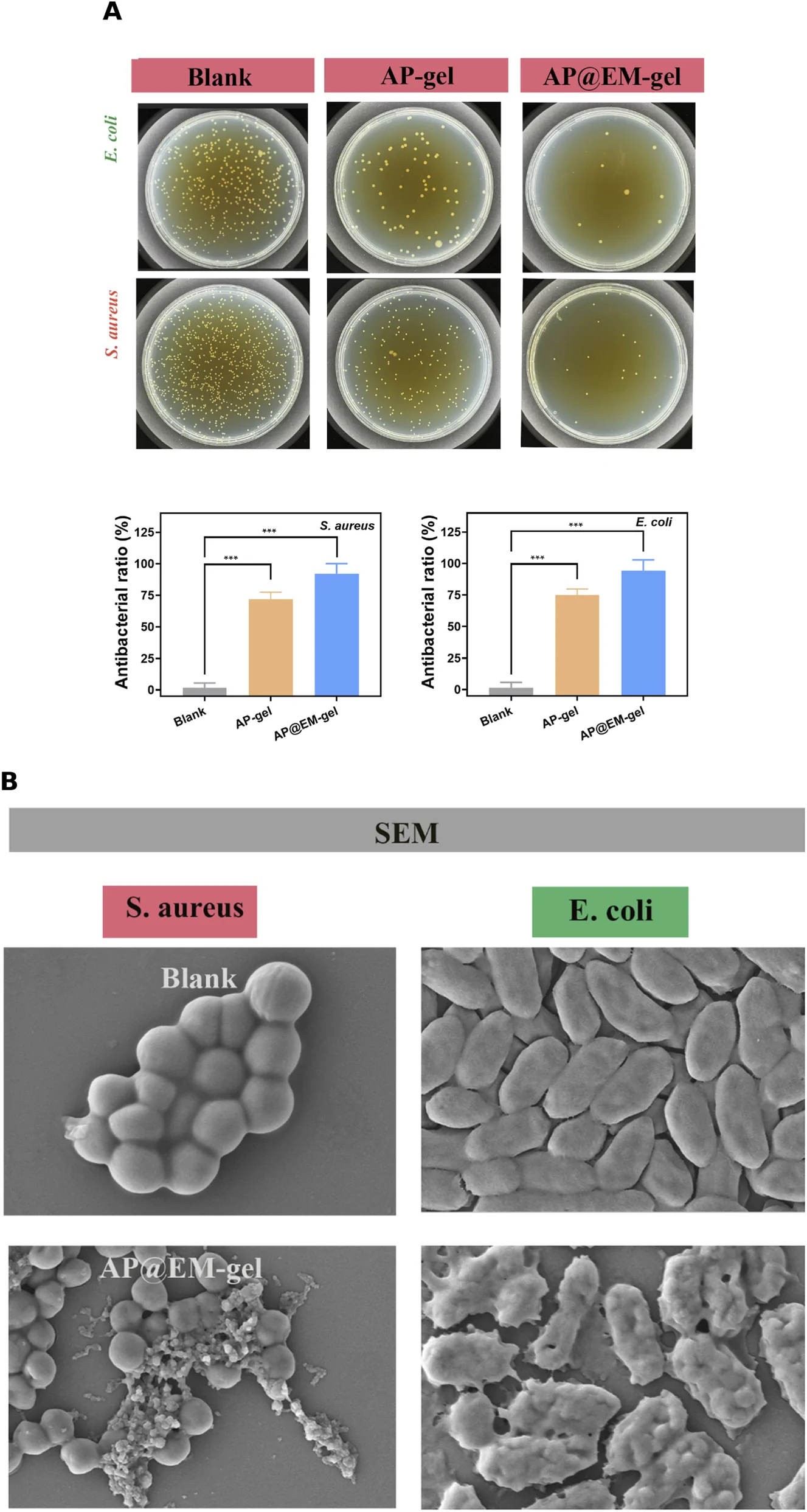
*In vitro* antibacterial performance. **(A)** Representative agar plates for *E. coli* and *S. aureus* (Blank, AP-gel, AP@EM-gel) with corresponding antibacterial-ratio quantification against *S. aureus* and *E. coli*. **(B)** SEM images of *S. aureus* and *E. coli* morphology after treatment (Blank and AP@EM-gel). Data are mean ± SD (n = 3). ***p < 0.001.

**FIGURE 5:**
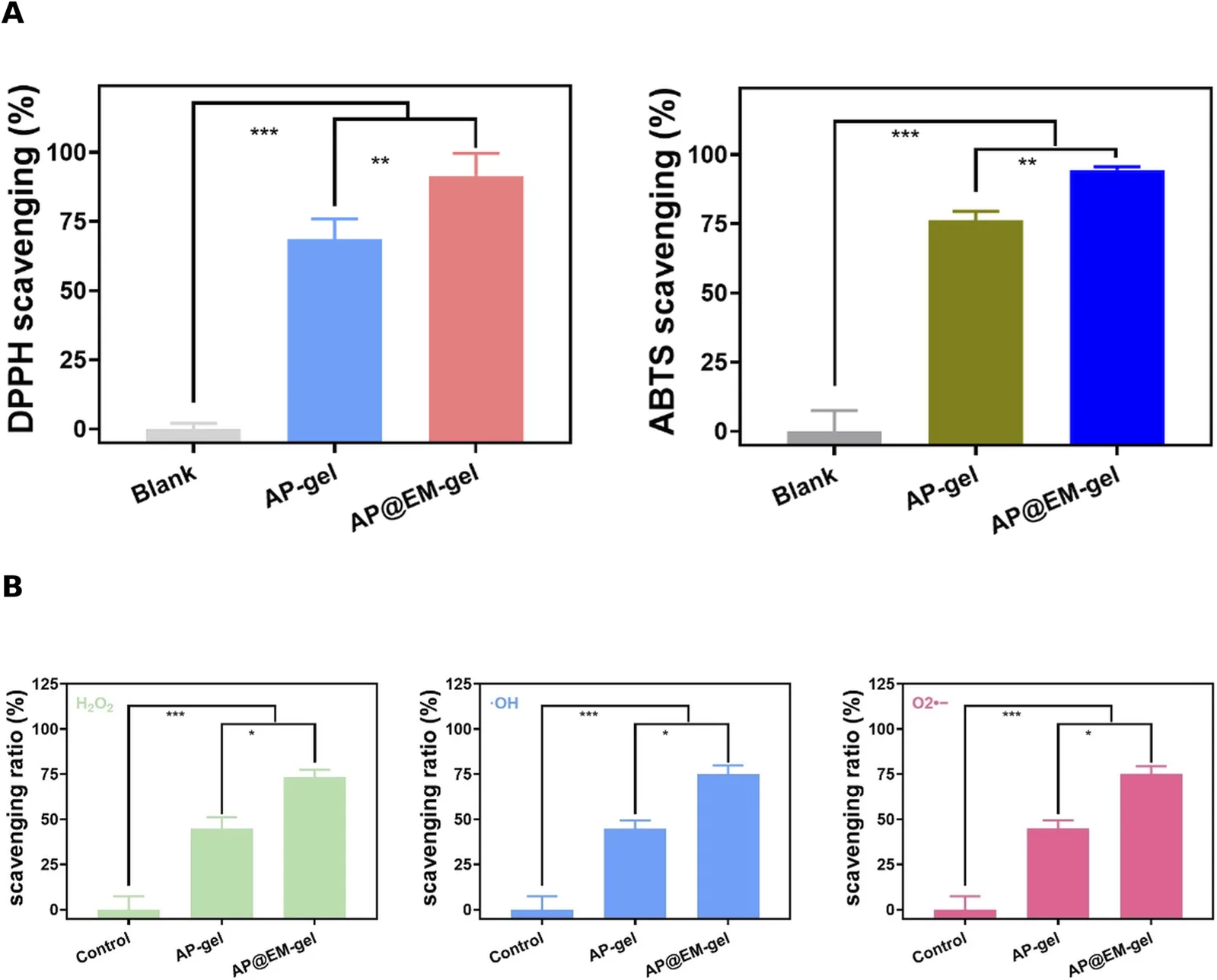
*In vitro* antioxidant performance. **(A)** DPPH and ABTS radical-scavenging assays. **(B)** Scavenging ratios of H_2_O_2_, ·OH and O_2_·^-^. Data are mean ± SD (n = 3). *p < 0.05, **p < 0.01, ***p < 0.001.

AP@EM-gel showed potent broad-spectrum antibacterial activity. After 3 h co-culture, colony counts fell sharply: the cationic EPBA scaffold (AP-gel) alone reduced viable bacteria by ≈ 70%, while AP@EM-gel achieved substantial bactericidal activity — ≈ 93% against *S. aureus* and ≈ 91% against *E. coli*—significantly exceeding AP-gel (***p < 0.001; Figure 4A). SEM confirmed the mechanism: bacteria exposed to AP@EM-gel lost their smooth, intact morphology and instead showed surface roughening, deep indentation, membrane rupture and leakage of intracellular contents, consistent with combined electrostatic (EPBA) and polyphenolic/ ROS-mediated (EGCG-MET) membrane disruption (Hyldgaard et al., 2014; Guo et al., 2018) (Figure 4B). Antioxidant testing across five assays demonstrated comprehensive, multi-species ROS neutralisation by AP@EM-gel: DPPH 91.4% and ABTS 94.5% (both significantly above AP-gel; Figure 5A), and 73.4%, 75.1%, and 75.3% scavenging of H_2_O_2_, ·OH and O_2_·^-^, respectively (Figure 5B) — far exceeding the Blank control and consistently superior to AP-gel.

**FIGURE 6:**
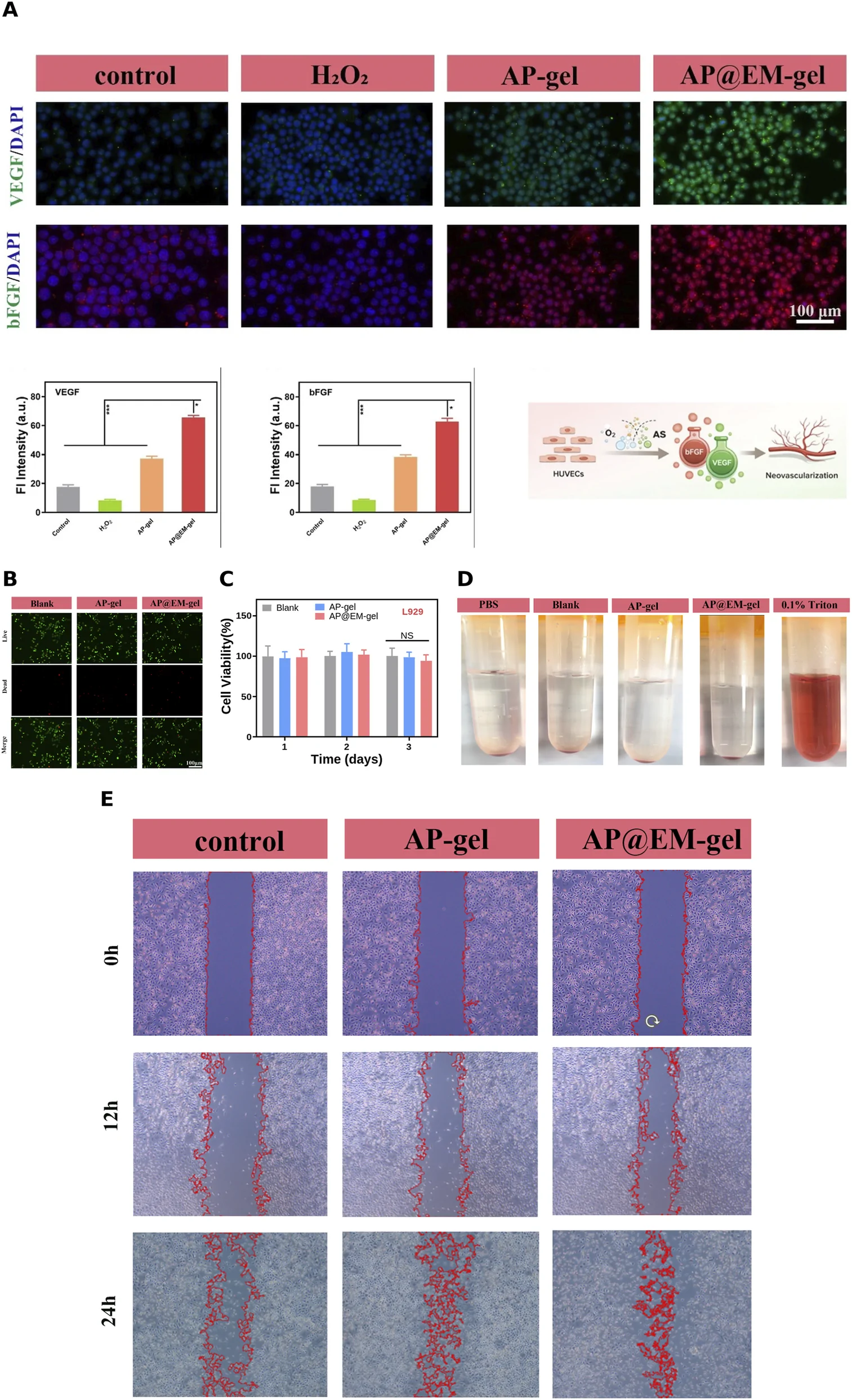
*In vitro* pro-angiogenic activity, cytocompatibility, haemocompatibility and migration. **(A)** VEGF (green)/bFGF (red) immunofluorescence in oxidatively stressed HUVECs (control, H_2_O_2_, AP-gel, AP@EM-gel; scale bar 100 μm), corresponding VEGF and bFGF fluorescence-intensity quantification, and a schematic of neovascularisation. **(B)** Live/dead staining of L929 cells. **(C)** CCK-8 viability of L929 cells over 3 days. **(D)** Erythrocyte haemolysis assay (PBS, Blank, AP-gel, AP@EM-gel, 0.1% Triton X-100). **(E)** Scratch-wound fibroblast migration at 0, 12, and 24 h. Data are mean ± SD (n = 3).

**FIGURE 7:**
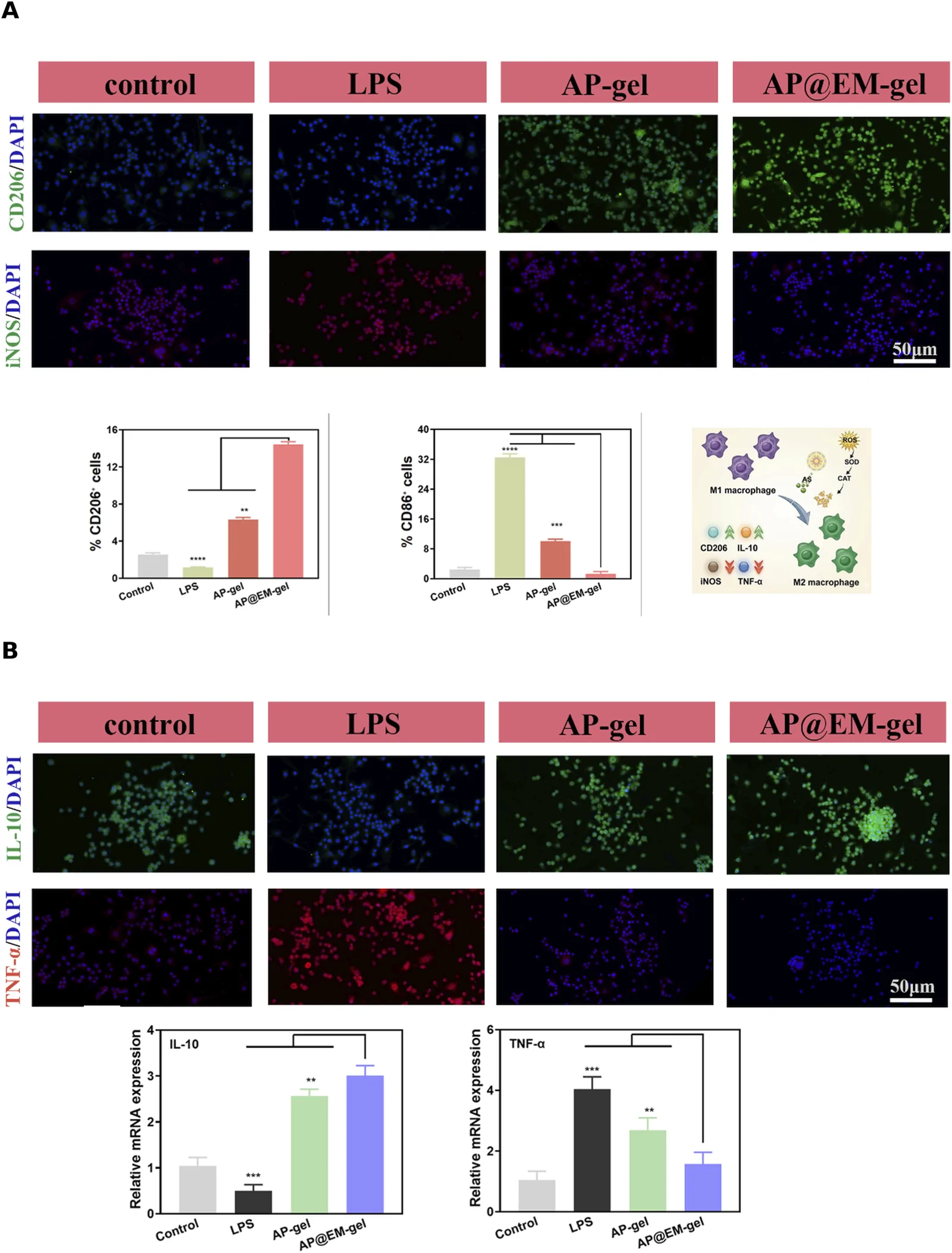
*In vitro* macrophage polarisation and cytokine regulation in LPS-stimulated RAW264.7 cells. **(A)** CD206 (green, M2)/iNOS (red, M1) immunofluorescence (control, LPS, AP-gel, AP@EM-gel; scale bar 50 μm), flow-cytometric CD206+ and CD86^+^ proportions, and a schematic of M1-to-M2 repolarisation. **(B)** IL-10/TNF-α immunofluorescence (scale bar 50 μm) and IL-10 and TNF-α mRNA expression by RT-qPCR. Data are mean ± SD (n = 3). *p < 0.05, **p < 0.01, ***p < 0.001, ****p < 0.0001.

The hydrogel was cytocompatible: live/dead staining showed predominantly viable (green) L929 fibroblasts with minimal cell death across Blank, AP-gel and AP@EM-gel groups (Figure 6B), consistent with CCK-8 viabilities above 90% (see Section 3.3). Under oxidative stress, AP@EM-gel restored angiogenic signalling: H_2_O_2_ markedly suppressed VEGF and bFGF in HUVECs, whereas AP@EM-gel produced the strongest recovery of both factors, significantly above AP-gel (Figure 6A). A scratch assay showed that both hydrogels supported fibroblast migration and faster gap closure than the control (Figure 6E). Finally, AP@EM-gel reprogrammed macrophage polarisation in an LPS-driven inflammatory model: CD206 (M2) immunofluorescence was strongest and iNOS (M1) weakest in the AP@EM-gel group (Figure 7A), and RT-qPCR confirmed the highest IL-10 and lowest TNF-α expression among all groups, a reciprocal pattern mirrored throughout the study (Figure 7B). Together these data establish that AP@EM-gel simultaneously controls infection and oxidative stress while actively supporting endothelial and reparative-immune functions.

### 3.3 Quantitative cytocompatibility, haemocompatibility and macrophage repolarisation

CCK-8 confirmed that neither AP-gel nor AP@EM-gel reduced L929 viability over 3 days, which remained above 90% at all concentrations with no significant difference from control (p > 0.05; Figure 6C). Haemolysis remained well below the 5% ISO 10993–4 safety threshold for all hydrogel groups, with no significant difference among Blank, AP-gel and AP@EM-gel and a large difference from the Triton X-100 positive control (***p < 0.0001; Figure 6D), confirming excellent haemocompatibility. Flow cytometry quantified the macrophage shift: AP@EM-gel produced the highest CD206+ (M2) proportion (14.4%; **p < 0.01 vs. LPS) and the lowest CD86+ (M1) proportion (1.2%) (Figure 7A), indicating the most pronounced repolarisation toward the reparative phenotype among all groups.

### 3.4 *In vivo* wound closure and early bacterial clearance

**FIGURE 8:**
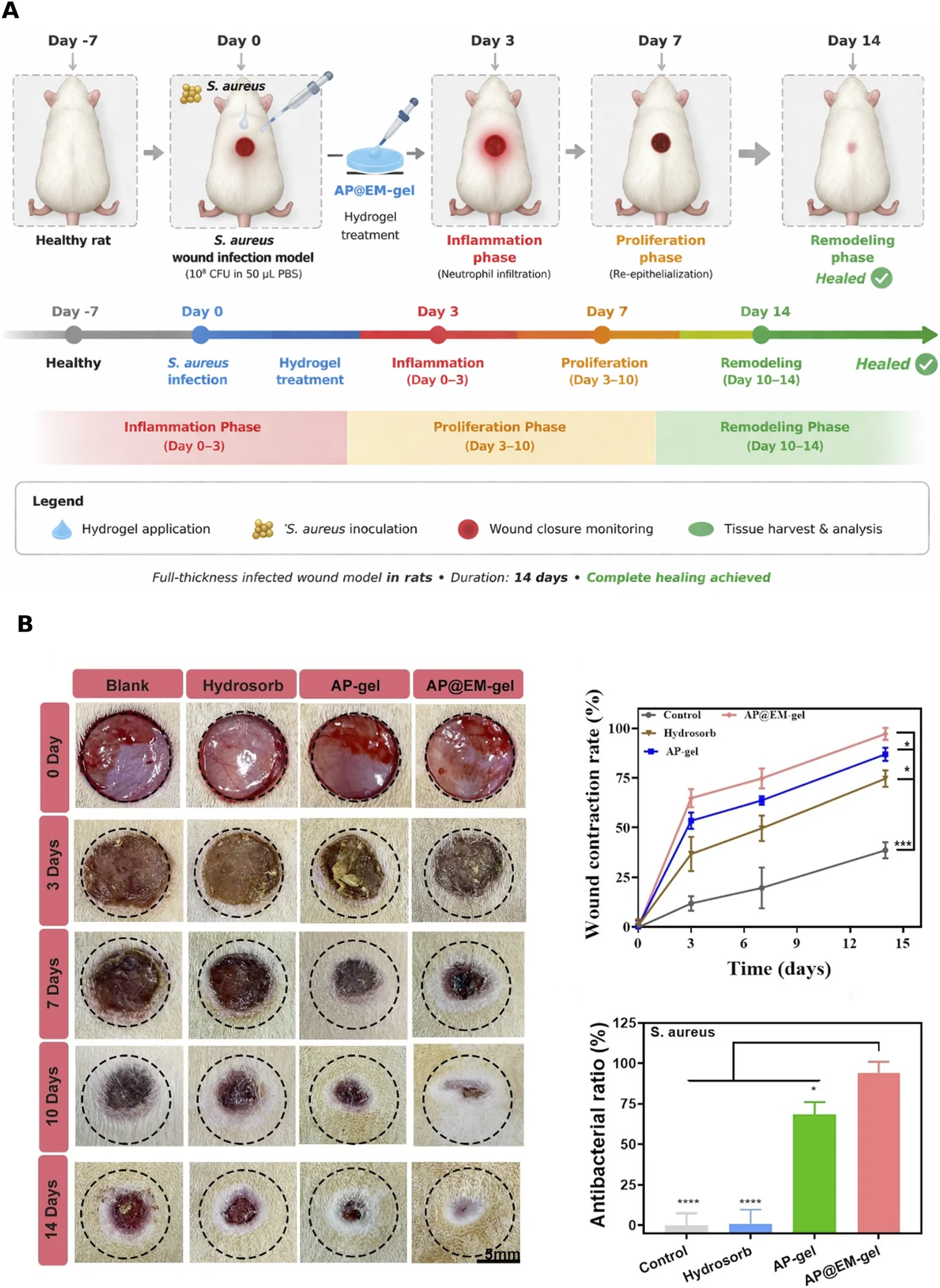
*In vivo* wound healing in the diabetic infected wound model. **(A)** Schematic of the experimental design and treatment timeline (inflammation day 0–3, proliferation day 3–10, remodelling day 10–14). **(B)** Representative wound photographs (Blank, Hydrosorb®, AP-gel, AP@EM-gel) at days 0, 3, 7, 10, and 14 (scale bar: 5 mm), wound-contraction rate over time, and day-3 antibacterial ratio against *S. aureus*. Data are mean ± SD (n = 6). *p < 0.05, ***p < 0.001, ****p < 0.0001.

In the STZ-induced diabetic S. aureus-infected wound model, all wounds were comparable at day 0, but the groups diverged rapidly thereafter (Figure 8). AP@EM-gel accelerated closure from the early inflammatory phase: by day 3 its contraction already exceeded the Blank group (*p < 0.05), and the advantage widened through the proliferative and remodelling phases. By day 14, AP@EM-gel achieved near-complete closure (≈100%) with a healed appearance resembling normal skin, compared with ≈ 80% for AP-gel, ≈ 75% for Hydrosorb® and only ≈ 38% for the untreated Blank group (*p < 0.05). Day-3 tissue CFU counts confirmed that this benefit was underpinned by early infection control: AP@EM-gel markedly reduced the wound bacterial burden, corresponding to an *in vivo* antibacterial rate of ≈ 100% relative to the Blank group (calculated from tissue CFU counts), AP-gel produced an intermediate ≈ 70% reduction, while Hydrosorb® and Blank showed essentially no antibacterial effect (****p < 0.0001 vs. AP@EM-gel), confirming that passive commercial dressings cannot control an established wound infection.

### 3.5 Histological wound quality: re-epithelialisation and collagen remodelling

**FIGURE 9:**
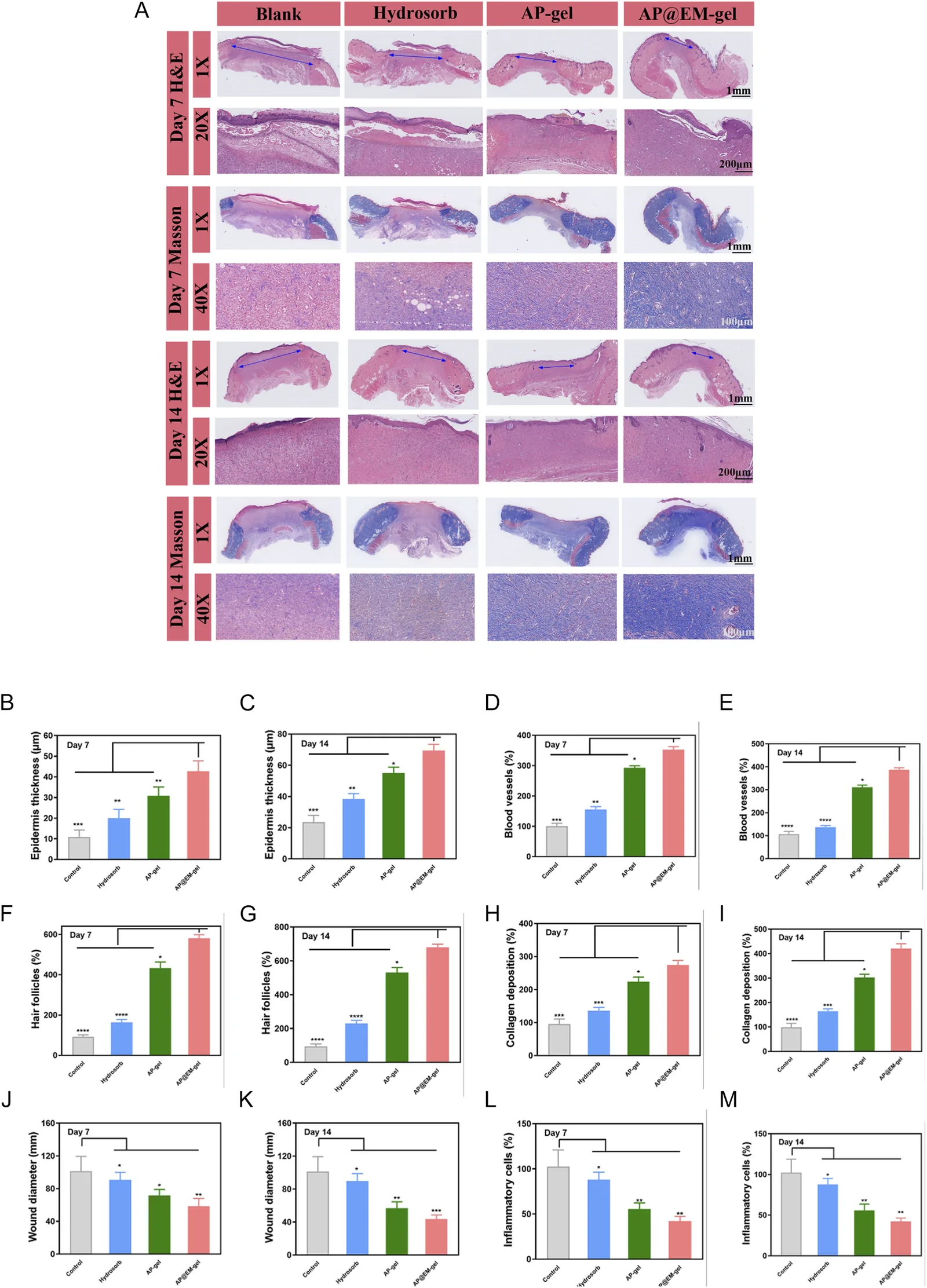
Histological evaluation of wound tissue at days 7 and 14. **(A)** Representative H&E (1× and 20×) and Masson’s trichrome (1× and 40×) staining (Blank, Hydrosorb®, AP-gel, AP@EM-gel); blue staining indicates collagen and blue double-headed arrows indicate wound diameter. Quantification of epidermal thickness at day 7 **(B)** and day 14 **(C)**, blood vessels at day 7 **(D)** and day 14 **(E)**, hair follicles at day 7 **(F)** and day 14 **(G)**, collagen deposition at day 7 **(H)** and day 14 **(I)**, wound diameter at day 7 **(J)** and day 14 **(K)**, and inflammatory cells at day 7 **(L)** and day 14 **(M)**. Data are mean ± SD (n = 6). *p < 0.05, **p < 0.01, ***p < 0.001, ****p < 0.0001.

H&E and Masson’s trichrome staining at days 7 and 14 showed that AP@EM-gel improved every measured dimension of tissue repair (Figure 9). AP@EM-gel produced the smallest wound diameter at both time points (day 7, **p < 0.01; day 14, ***p < 0.001 vs. Blank), the greatest epidermal thickness with a continuous, structurally regular neo-epidermis, and the most abundant hair-follicle regeneration—a sensitive indicator of true regenerative rather than scar-based repair—significantly exceeding all other groups. Inflammatory-cell infiltration was lowest in the AP@EM-gel group at both time points (**p < 0.01 vs. Blank), indicating an earlier and more complete transition out of the inflammatory phase. Masson’s trichrome showed that AP@EM-gel drove the greatest collagen deposition; at day 14 its collagen fibres were dense, thick and well aligned, approaching the architecture of normal dermis, whereas Blank and Hydrosorb® wounds remained sparse and disorganised.

### 3.6 Wound angiogenesis and vascular maturation

**FIGURE 10:**
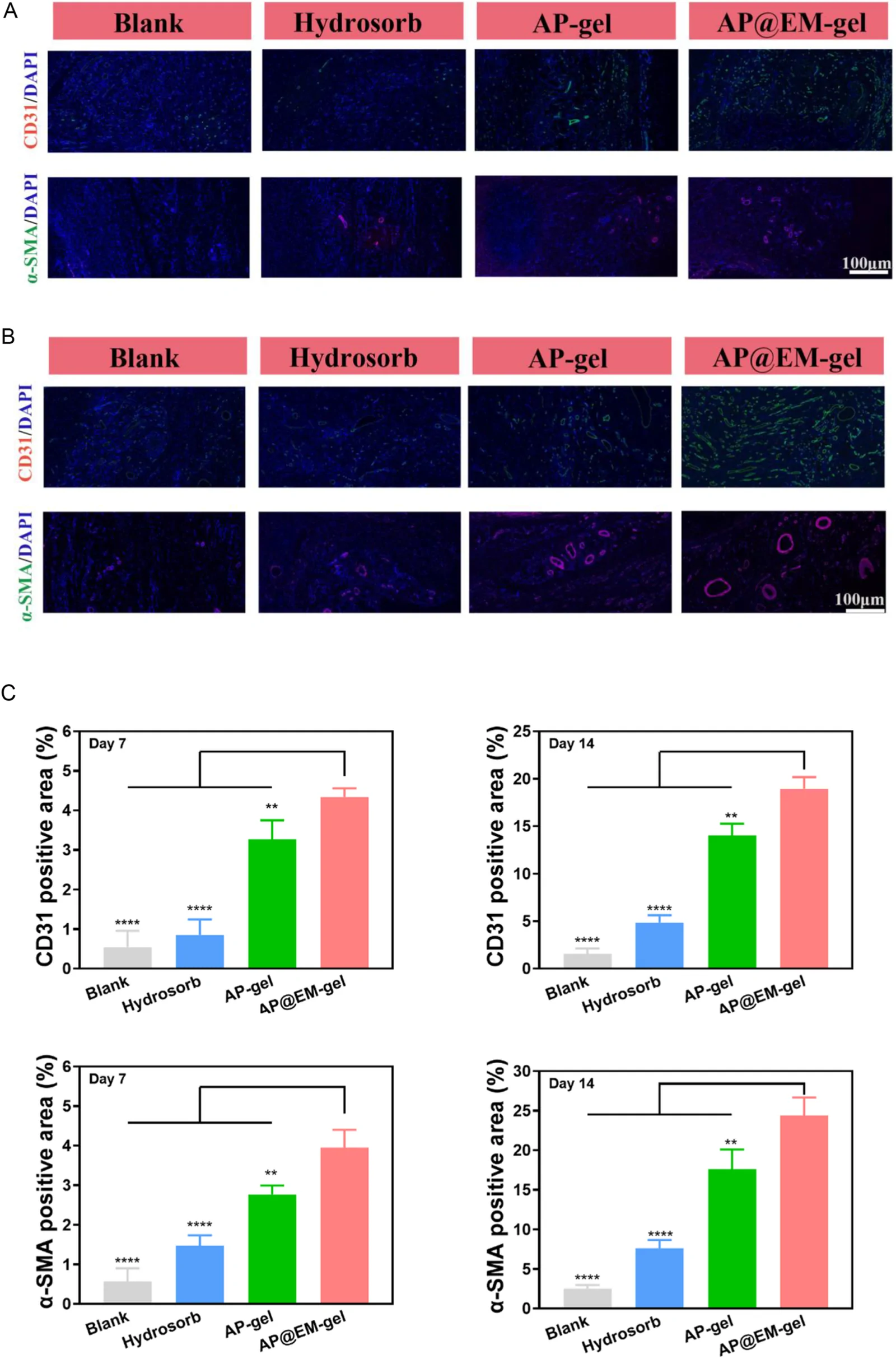
**(A)** *In vivo* angiogenesis and vascular maturation at day 7. High-resolution CD31 (green)/DAPI (blue) and α-SMA (magenta)/DAPI immunofluorescence of wound tissue from the Blank, Hydrosorb®, AP-gel and AP@EM-gel groups. Scale bar: 100 μm. **(B)** *In vivo* angiogenesis and vascular maturation at day 14. High-resolution CD31 (green)/DAPI (blue) and α-SMA (magenta)/DAPI immunofluorescence of wound tissue from the Blank, Hydrosorb®, AP-gel and AP@EM-gel groups. Scale bar: 100 μm. **(C)** Quantification of angiogenesis and vascular maturation. CD31^+^ area at day 7 and day 14, followed by α-SMA + area at day 7 and day 14, arranged from top left to bottom right. Data are mean ± SD (n = 6). **p < 0.01, ****p < 0.0001.

Dual CD31 (endothelium)/ α-SMA (pericyte/ smooth muscle) immunofluorescence showed that AP@EM-gel substantially enhanced both the quantity and the maturity of new vessels (Figures 10A–C). Untreated and Hydrosorb®-treated wounds displayed sparse, poorly formed CD31+ structures and minimal α-SMA, consistent with the impaired angiogenesis typical of diabetic wounds. AP@EM-gel produced the strongest CD31 and α-SMA signals with abundant, well-defined vascular structures at both day 7 and day 14, with CD31+ and α-SMA + areas significantly greater than all comparator groups (**p < 0.01 vs. AP-gel; ****p < 0.0001 vs. Blank and Hydrosorb®). The co-localisation of endothelial and pericyte markers indicated the formation of structurally mature, stabilised vasculature rather than transient capillary sprouts, in agreement with the *in vitro* restoration of VEGF and bFGF.

### 3.7 *In vivo* inflammatory microenvironment: IL-10 and TNF-α

**FIGURE 11:**
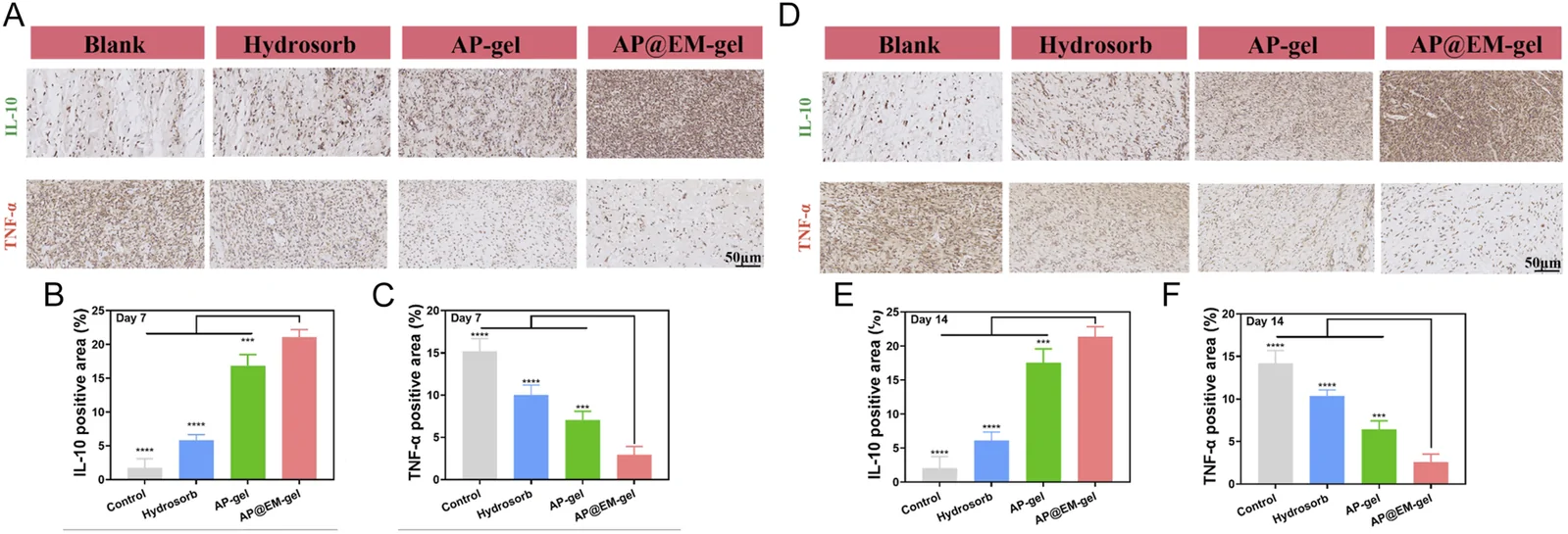
*In vivo* inflammatory cytokines in wound tissue. **(A)** Day-7 IL-10 (upper) and TNF-α (lower) immunohistochemistry (brown, positive; scale bar 50 μm), with quantification of **(B)** IL-10+ and **(C)** TNF-α+ areas. **(D)** Day-14 IL-10 and TNF-α immunohistochemistry, with quantification of **(E)** IL-10+ and **(F)** TNF-α+ areas. Data are mean ± SD (n = 6). ***p < 0.001, ****p < 0.0001.

Immunohistochemistry confirmed that AP@EM-gel reciprocally rebalanced the wound cytokine milieu at the protein level (Figure 11). Untreated wounds showed the lowest anti-inflammatory IL-10 and the highest pro-inflammatory TNF-α staining at both day 7 and day 14, indicating sustained inflammatory activation. AP@EM-gel produced the strongest IL-10 and the weakest TNF-α signals among all groups, with both differences highly significant relative to Blank (****p < 0.0001 at day 7; IL-10 ***p < 0.001 and TNF-α ****p < 0.0001 at day 14). These tissue-level findings closely mirrored the *in vitro* RT-qPCR and immunofluorescence results, confirming durable reciprocal regulation of inflammation throughout the healing period.

### 3.8 *In vivo* macrophage repolarisation toward the M2 phenotype

**FIGURE 12:**
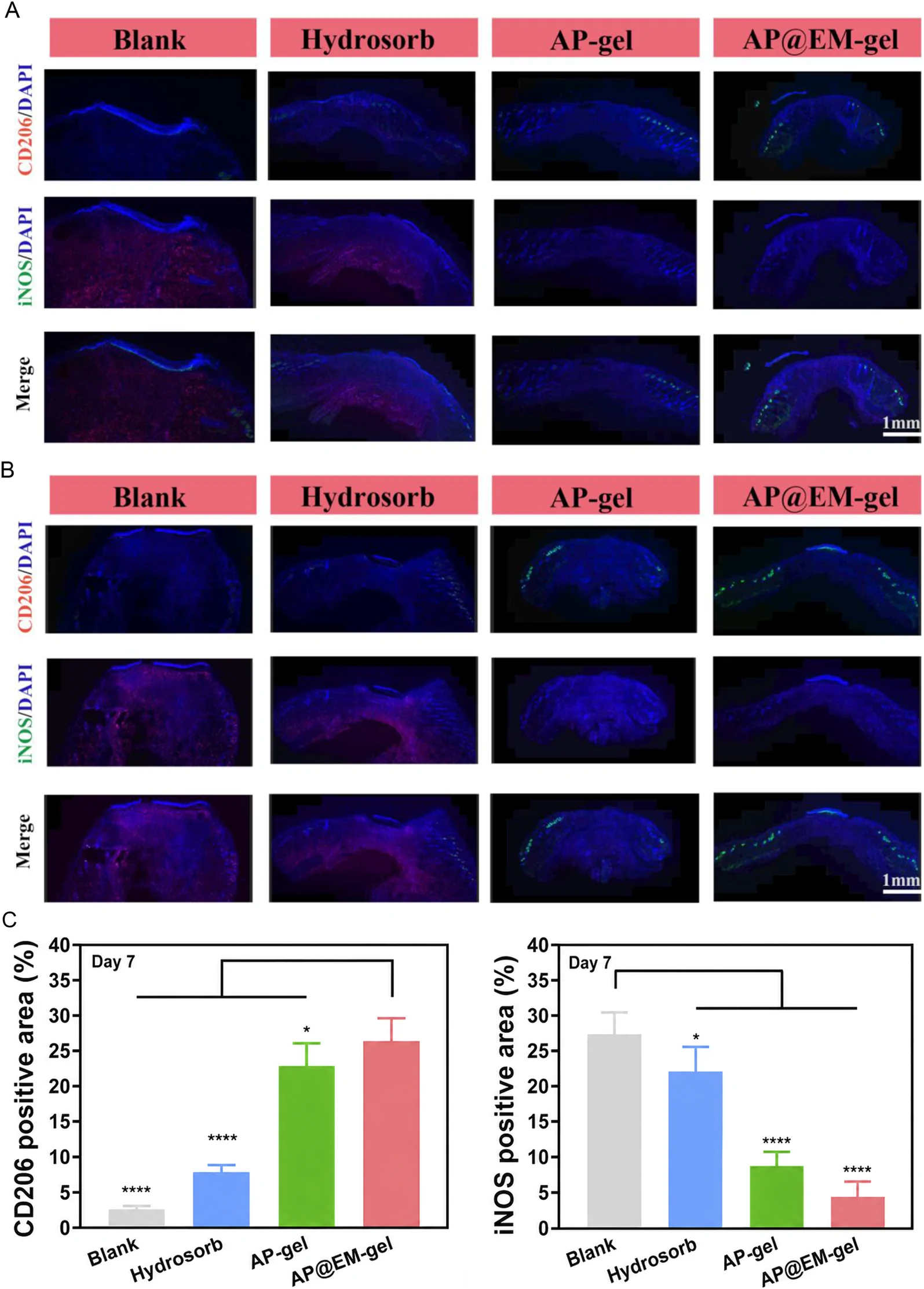
**(A)** *In vivo* macrophage polarisation at day 7. High-resolution CD206/iNOS/merged immunofluorescence of wound tissue from the Blank, Hydrosorb®, AP-gel and AP@EM-gel groups (blue, DAPI). Scale bar: 1 mm. **(B)** *In vivo* macrophage polarisation at day 14. High-resolution CD206/iNOS/merged immunofluorescence of wound tissue from the Blank, Hydrosorb®, AP-gel and AP@EM-gel groups (blue, DAPI). Scale bar: 1 mm. **(C)** Quantification of macrophage polarisation at day 7. CD206+ area (left) and iNOS + area (right). Data are mean ± SD (n = 6). *p < 0.05, ****p < 0.0001.

CD206 (M2)/ iNOS (M1) immunofluorescence of wound tissue confirmed that the macrophage reprogramming observed *in vitro* operated *in vivo* (Figures 12A–C). Untreated and Hydrosorb® wounds remained iNOS-dominant with minimal CD206 at both day 7 and day 14, reflecting persistent M1 inflammation. AP@EM-gel produced the strongest CD206 and weakest iNOS signals at both time points; quantitatively, at day 7 it showed the highest CD206+ area (****p < 0.0001 vs. Blank and Hydrosorb®; *p < 0.05 vs. AP-gel) and the lowest iNOS + area among all groups, while the representative day-14 immunofluorescence images indicate that this M2-dominant pattern was maintained into the remodelling phase. This sustained M2 dominance provides a coherent mechanistic explanation for the superior angiogenesis, collagen organisation and re-epithelialisation observed in the AP@EM-gel group.

## 4 Discussion

The central design premise of this work is that diabetic wound chronicity is a network pathology, in which infection, oxidative stress, M1-dominated inflammation and impaired angiogenesis reinforce one another, and that durable repair therefore requires a dressing able to act at several nodes of this network simultaneously. AP@EM-gel was built to do exactly this, and the convergent evidence presented here indicates that it succeeds. Each component contributes a distinct and complementary function: the Alg-DA/EPBA boronate-ester network supplies injectability, self-healing, wet-tissue adhesion and pathology-gated release; the cationic EPBA backbone provides intrinsic, resistance-independent antibacterial activity (Hyldgaard et al., 2014; Yang et al., 2021); and the EGCG-MET nanoparticles deliver the antioxidant, anti-inflammatory and pro-angiogenic payload (Mokra et al., 2022), an approach consistent with co-assembled polyphenol–metformin nanoparticle systems reported elsewhere (Da et al., 2025). The repeated superiority of AP@EM-gel over the drug-free AP-gel across antibacterial, antioxidant, angiogenic, immunomodulatory and *in vivo* endpoints confirms that the EGCG-MET payload makes an independent and quantitatively important contribution, validating the multi-component strategy rather than attributing the benefit to the scaffold alone.

A defining feature of the platform is microenvironment-responsive delivery. Because boronate-ester bonds hydrolyse under both acidic and oxidative conditions, AP@EM-gel released markedly more EGCG and MET as pH fell and H_2_O_2_ rose, reaching maximal release under the combined pathological condition that characterises the most severely infected wound regions. This converts the wound’s own biochemical abnormalities into a release trigger, so that drug availability tracks pathological severity in space and time—an adaptive behaviour that passive or constantly leaking systems cannot reproduce (Haidari et al., 2022; Yu et al., 2024). The same logic links the material design to the biology: the early, day-3 divergence in wound closure and bacterial clearance is consistent with rapid drug release under the acute low-pH, high-ROS conditions immediately after infection (Zheng et al., 2022), while the sustained anti-inflammatory and pro-angiogenic effects through day 14 reflect continued, severity-matched delivery as the wound evolves.

Mechanistically, the antibacterial action combines EPBA-mediated electrostatic membrane disruption with polyphenolic membrane intercalation and ROS-amplified damage from EGCG-MET, a dual, membrane-active mode of action consistent with the SEM evidence of membrane rupture and content leakage (Wang et al., 2020; Liu et al., 2022). Because this mechanism targets conserved physical properties of the bacterial envelope rather than a specific molecular target, it is intrinsically less vulnerable to classical resistance—an attractive property for chronic diabetic wounds, where resistant and polymicrobial infections are common (Afonso et al., 2021; Fan et al., 2024). The antioxidant action is similarly multi-pronged: EGCG provides immediate hydrogen-atom and electron-transfer radical quenching and Fe^2+^ chelation that blocks Fenton chemistry, and may induce endogenous antioxidant enzymes through Nrf2/HO-1 signalling (Capasso et al., 2025; Tajammal et al., 2025), while metformin may reduce inflammatory and oxidative injury through AMPK-associated metabolic regulation and suppression of NLRP3 inflammasome activation (Saisho, 2015; Yang et al., 2019; Wei et al., 2025); these specific pathways were inferred from the literature rather than directly assessed here. The breadth of scavenging across five chemically distinct assays indicates that this is genuine multi-species neutralisation rather than assay-specific activity, which matters because diabetic wounds generate the full spectrum of ROS simultaneously (Dunn et al., 2015; Deng et al., 2021), and is comparable to recently reported EGCG-based antioxidant wound dressings (Sun et al., 2025).

The immunological findings are arguably the mechanistic pivot of the study. Chronic wounds fail not merely because pathogens or ROS are present but because the macrophage compartment cannot complete the M1-to-M2 transition required to enter the proliferative phase (Lin et al., 2022; Clayton et al., 2024). By increasing CD206 and IL-10 while suppressing iNOS, CD86 and TNF-α at phenotypic, quantitative and transcriptional levels *in vitro*—and by maintaining M2 dominance *in vivo* from day 7 to day 14 — AP@EM-gel demonstrates that it actively redirects wound immunity toward repair. This is biologically consequential because M2 macrophages secrete TGF-β, VEGF and IGF-1, providing a direct causal route from immunomodulation to the enhanced collagen synthesis, vascular maturation and re-epithelialisation documented histologically; metformin may further reinforce this anti-inflammatory shift through AMPK-dependent NLRP3 inflammasome suppression (Saisho, 2015; Yang et al., 2019; Alvarez et al., 2016; Qian et al., 2022). The recovery of VEGF and bFGF in oxidatively stressed endothelial cells and the dense, pericyte-stabilised CD31/α-SMA vasculature *in vivo* are coherent with this immuno-angiogenic axis and with the known HIF-1α/VEGF and AMPK/eNOS activities of EGCG and metformin (Martin-Montalvo et al., 2013; Zheng et al., 2023).

The comparison with Hydrosorb® is important for interpreting clinical relevance. Hydrosorb® is a genuine, clinically used dressing rather than a sham control, yet it showed negligible antibacterial activity, failed to promote M2 polarisation or suppress TNF-α, and gave substantially inferior closure, vascularisation and tissue quality. This underscores that passive moisture management—even when commercially validated—is insufficient against the multifactorial pathology of an infected diabetic wound, and that the active, multi-target strategy embodied by AP@EM-gel addresses precisely the gap that motivated the study and aligns with the most effective recent multifunctional diabetic-wound hydrogels (Monaghan et al., 2023; Zhao et al., 2024; Yao et al., 2022; Zhang et al., 2025). A further strength of the evidence is methodological: the *in vivo* conclusion rests on the convergence of five independent histological and molecular readouts, all identifying AP@EM-gel as superior, which guards against single-endpoint artefacts and against the well-known pitfall of equating surface closure with quality of repair.

Several limitations should temper these conclusions. The antibacterial work used planktonic cultures of two reference strains rather than mature biofilms or characterised resistant clinical isolates; the *in vivo* study used a single rodent species over 14 days; and detailed degradation, long-term stability and large-animal safety data remain to be established. From a translational standpoint, the scalability and batch-to-batch reproducibility of the EGCG–MET co-assembly, together with the shelf-life and storage stability of the pre-formed gel, will also need to be defined before clinical development. Future work should therefore extend pathogen and biofilm validation, lengthen the observation window, test larger-animal models, and dissect the relative contributions of EGCG and metformin through component-omission and pathway-inhibition studies. Nonetheless, the present data provide a coherent, mechanistically grounded and clinically relevant case that AP@EM-gel interrupts the chronic-wound pathological loop at multiple nodes simultaneously.

## 5 Conclusion

We developed AP@EM-gel, an injectable, self-healing, pH/ROS dual-responsive nanocomposite hydrogel that co-delivers epigallocatechin gallate and metformin from a dopamine-alginate/phenylboronic-acid-polylysine network. The platform releases its payload in proportion to wound acidity and oxidative stress and combines broad-spectrum antibacterial activity, comprehensive multi-species ROS scavenging, excellent cyto- and haemocompatibility, restoration of endothelial pro-angiogenic signalling, and robust reprogramming of macrophages toward the reparative M2 phenotype. In a diabetic, S. aureus-infected full-thickness wound model it accelerated closure to near-completion and outperformed a commercial dressing across all measured endpoints—bacterial clearance, re-epithelialisation, collagen organisation, angiogenesis and inflammatory resolution. By engaging infection, oxidative stress, inflammation and impaired angiogenesis simultaneously, AP@EM-gel offers a mechanistically coherent, multi-target strategy and a promising candidate for next-generation smart dressings in diabetic chronic wound management, although confirmation in larger-animal models and over longer observation windows will be required before clinical translation.

## Data Availability

The original contributions presented in the study are included in the article/supplementary material, further inquiries can be directed to the corresponding author.
